# OCT Angiography Findings in Macula-ON and Macula-OFF Rhegmatogenous Retinal Detachment: A Prospective Study

**DOI:** 10.3390/jcm9123982

**Published:** 2020-12-09

**Authors:** Francesco Barca, Daniela Bacherini, Francesco Dragotto, Ruggero Tartaro, Chiara Lenzetti, Lucia Finocchio, Gianni Virgili, Tomaso Caporossi, Fabrizio Giansanti, Alfonso Savastano, Stanislao Rizzo

**Affiliations:** 1 Department of Neurosciences, Psychology, Drug Research and Child Health Eye Clinic, University of Florence, AOU Careggi, 50139 Firenze, Italy; barcaf@hotmail.com (F.B.); daniela.bacherini@gmail.com (D.B.); ruggerotartaro@yahoo.it (R.T.); chiaralenze@gmail.com (C.L.); luciafinocchio@gmail.com (L.F.); gianni.virgili@unifi.it (G.V.); tomaso.caporossi@me.com (T.C.); fabrizio.giansanti@unifi.it (F.G.); 2 Department Head, Neck and Sensory Organs, Ophthalmology, University Cattolica del Sacro Cuore, 00168 Rome, Italy; asavastano21@gmail.com (A.S.); stanislao.rizzo@gmail.com (S.R.)

**Keywords:** retinal detachment, vitrectomy, scleral buckle, rhegmatogenous retinal detachment, optical coherence tomography angiography, optical coherence tomography

## Abstract

**Background:** The aim of the study was to evaluate pre-operative and post-operative retinal vasculature using optical coherence tomography angiography (OCTA) in patients who underwent rhegmatogenous retinal detachment (RRD) surgery repair. **Materials and Methods:** A total of 33 eyes were included in this prospective consecutive observational study: 15 affected by macula-ON and 18 by macula-OFF RRD. Superficial (SCP), deep capillary plexus (DCP), and foveal avascular zone (FAZ) area variations were evaluated by OCTA and correlated with visual acuity (VA) during a six-month follow-up. **Results:** In the macula-ON group, the preoperative vascular density (VD) of the whole SCP (wSCP) on affected eyes was lower than that of the fellow eyes (*p* < 0.05); this difference disappeared at 6 months after surgery (*p* = 0.88). The wSCP VD and the parafoveal SCP (pfSCP) VD increased during follow-up (*p* < 0.05); moreover, the higher the preoperative wSCP and pfSCP VD, the better the baseline VA (*p* < 0.05). In the macula-OFF group, at the first and sixth months after surgery, the larger the FAZ, the lower the VA (*p* < 0.05). **Conclusions:** Macula-ON SCP VD affected preoperative VA, and it was lower than the fellow eye, but recovered over time. In the macula-OFF group, a larger FAZ area was related to a worse VA, as is the case in diabetes and in retinal vein occlusion (RVO).

## 1. Introduction

Rhegmatogenous retinal detachment (RRD) is the separation of the retinal neuroepithelium from the retinal pigmented epithelium due to the presence of subretinal fluid (SRF), which infiltrates under the retina, through a retinal tear. It could cause a dramatic visual acuity reduction if not rapidly detected and treated. However, despite the high anatomical success ratio, the postoperative visual acuity (VA) gain often results incomplete. The advent of the optical coherence tomography (OCT) technologies has allowed us to visualize, in a non-invasive way, ultrastructural details of the macular and retinal anatomy related to the presence of the RRD before and after surgery that can be responsible of this incomplete VA gain.

Studies [1,2,3] based on OCT reported different morphologies and complication of retinal reattachment correlated to a lower best-corrected visual acuity (BCVA), a cystoid macular edema (CME), persistent subretinal fluid (PSF), the formation of epiretinal macular membranes (ERM), and the disruption of the inner segment/outer segment (IS/OS). In PPV surgery, current studies relate the possible role of retinal shift on visual acuity [4].

Optical coherence tomography angiography (OCTA) is a non-invasive tool capable of providing qualitative and quantitative analysis of retinal and choroidal microvasculature; it may be used to evaluate the macular vasculature and its modification caused by RRD that should contribute to a lower VA gain after RRD surgery.

Therefore, in our study, OCTA was used to evaluate pre-operative and post-operative changes in the retinal vasculature in patients affected by RRD. The retinal deep (DCP) and superficial (SCP) capillary plexus, and the foveal avascular zone (FAZ) area have been evaluated and correlated with patients’ postoperative functional recovery by means of BCVA.

## 2. Experimental Section

We conducted a prospective consecutive observational study evaluating structural changes before and after surgery for RRD, using OCTA.

The study was conducted at the eye clinic of Azienda Ospedaliero-Universitaria di Careggi, Florence. This study adhered to the tenets of the current version of the Declaration of Helsinki (52nd WMA General Assembly, Edinburgh, Scotland, UK, October 2000), and written informed consent was obtained from all patients prior to participation in the study. Approval from The Institutional Review Board/Ethics Committee was obtained.

Patients who underwent pars plana vitrectomy (PPV) or scleral buckle (SB) for primary uncomplicated RRD between January 2017 and April 2017 were evaluated for inclusion in the study. Only recent onset RRD (duration less than 7 days from symptom onset) were included. Surgery was performed within 72 h from diagnosis.

To make data comparable, only patients affected by simple or moderate primary RRD [5], with the absence of other underlying ocular pathologies, were enrolled. They were divided into two groups on the basis of macular status before surgery: patients without macular involvement were included in the group “macula-ON”, while patients with macular involvement were included in the group “macula-OFF”. Subjects with pre-existing or previous ophthalmological diseases, pathologic myopia (<−6D), proliferative vitreoretinopathy, or previous vitreoretinal surgery were excluded. Only patients with pre-operative OCT and OCTA images of adequate quality were included. Patients undergoing silicone oil injection during surgery, or with retained intraocular gas after surgery or postoperative media opacities were excluded because of inadequate OCTA acquisition and low reliability of vascular density measurement.

Additionally excluded from this analysis were patients with aphakia, proliferative retinal vascular disease, endophthalmitis, presence of intraocular foreign body, giant retinal tears, precipitating ocular trauma, prior posterior uveitis, other intraocular surgery within 90 days, or dense vitreous hemorrhage (2+ or greater).

All the patients underwent a complete ophthalmological examination of the affected and the fellow eye including BCVA measurement using ETDRS (Early Treatment Diabetic Retinopathy Study) 2-m charts and wide-field retinography (Daytona, Optos plc, Dunfermline, Scotland, UK), before and 1, 3, and 6 months after surgery. Axial length (AL) measurement was obtained using noncontact partial coherence laser interferometry (IOL Master, version 3.01; Carl Zeiss Meditec, Jena, Germany) if macula was attached, or immersion ultrasound biometry (Quantel Compact Touch, Quantel Medical, TX, USA) if not; eyes with AL > 26 mm were considered as highly myopic. In the macula-ON patients, OCTA centered in the macular area (RTVue XR Avanti, Optovue, Fremont, CA, USA) was performed in both eyes before the operation and 1, 3, and 6 months after surgery. For each eye, a 3 mm × 3 mm fovea-centered OCTA scan was performed. The analysis was conducted on the superficial capillary plexus (SCP) and the deep capillary plexus (DCP); the vascular density was detected using Optovue’s AngioAnalytics software (Optovue, Inc., Fremont, CA, USA), investigating the whole superficial, the superficial parafoveal, the superficial foveal, the whole deep, the deep parafoveal, and the deep foveal plexi. Moreover, the foveal avascular zone (FAZ) area was automatically calculated.

In all the cases, the correct automatic segmentation of the plexi was checked before proceeding with the vascular density analysis. Manual adjustment of the segmentation was performed in cases of alteration of the macular cytoarchitecture.

Central macular thickness (CMT) was assessed by the same OCT system at the same time as the retinal vasculature using the retina map mode.

The OCTA acquisitions in patients affected by macula off RRD before surgery were not taken into consideration because, due to the retinal detachment involving the macular region, the OCTA acquisition and layer segmentation were of inadequate quality and were not reliable for analysis. Measurements on the unaffected fellow eye in the same patients were conducted to find potential correlations between various parameters at baseline and during the follow up.

Three different authors observed and analyzed every OCTA image (F.D., D.B., F.B.).

All the patients underwent scleral buckling (SB) or pars plana vitrectomy (VPP) depending on the surgeon’s preference. All the procedures were performed under retrobulbar anesthesia by the same surgeon (S.R.). Standard three-port 25-gauge PPV was performed using the Alcon Constellation system (Alcon Laboratories, Inc., Fort Worth, TX, USA). After central and peripheral vitreous removal with 25-gauge incision, triamcinolone acetonide was injected to visualize residual posterior hyaloid, which should be removed completely. Complete fluid/air exchange was performed, followed by endophotocoagulation. Combined phacoemulsification, aspiration, and intraocular lens implantation were performed in cases of phakic patients.

The scleral buckling group underwent 360° scleral buckling and cryopexy of the tear areas; segmental radial or circumferential buckles were sutured above the retinal breaks, and external subretinal fluid (SRF) drainage was performed.

Patients were asked to maintain a prone posture for 7 days. Retinal reattachment was defined as the complete disappearance of subretinal fluid and flattening of the retinal breaks after gas absorption.

The quantitative changes analyzed using OCTA were FAZ area (mm^2^), and vascular density (VD) in the SCP and the VD-DCP (%), assessed through considering the whole, the foveal, and parafoveal areas.

Statistical analysis was performed using STATA software version 15.1 (StataCorp. College Station, TX, USA).

Descriptive statistics were used to summarize mean values and standard deviations of all the numerical data.

Pearson coefficient was used as a method to investigate the correlation between OCTA and demographic/clinical variables.

A *p*-value < 0.05 was considered statistically significant.

## 3. Results

We identified 96 patients affected by primary RRD successfully treated with a single procedure of PPV and SB between January 2017 and April 2017.

After considering inclusion and exclusion criteria, we enrolled 33 patients.

The baseline demographics and ocular characteristics of the enrolled patients are summarized in Table 1.

The interval between retinal detachment diagnosis and the operation was 1.48 ± 0.67 days.

Primary retinal reattachment was obtained in 100% of the patients, and no RRD recurrence was observed. No significant intraoperative or postoperative complications were observed, such as choroidal hemorrhage, subretinal hemorrhage, endophthalmitis, or proliferative vitreoretinopathy.

The clinical and OCTA parameters are summarized in Table 2 and Table 3.

### 3.1. Visual Acuity

In the macula-ON group, BCVA showed a statistically significant linear progression, with a significant improvement from the baseline (*p* < 0.001); similarly, in the macula OFF group, a significant BCVA improvement from the baseline was found (*p* < 0.001).

The BCVA progression showed a different trend in the two groups during follow-up—at 1 month, the macula-ON patients had a better VA in comparison with the macula-OFF group (*p* < 0.05). Six months after surgery, the VA differences between the two groups were not significative.

### 3.2. OCTA Findings

#### 3.2.1. “Macula On” Group

The preoperative VD of the whole SCP (wSCP) on affected eyes was proven to be significantly lower in comparison with the fellow eyes (*p* < 0.05); nevertheless, this difference between the two groups disappeared at 6 months after surgery (*p* = 0.88), with a restoration of the normal wSCP vascular density in the affected eyes. The VD of the wSCP in affected eye significantly increased during follow-up (*p* < 0.001); moreover, it was correlated to preoperative BCVA (*p* < 0.05)—the higher the preoperative wSCP vascular density, the better the baseline visual acuity.

**Figure 1:**
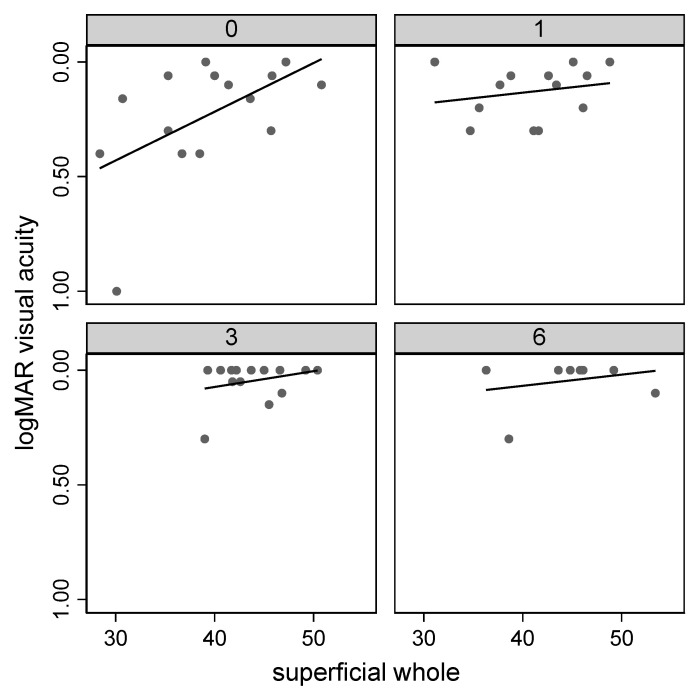
Correlation of the whole superficial capillary plexus vessel density (superficial whole) with visual acuity during follow-up.

The preoperative VD of the parafoveal SCP (pfSCP) showed a significant increase during follow-up (*p* < 0.05), and a linear correlation between VD pfSCP and BCVA was found pre-operatively (*p* < 0.05, R = −0.5357) (Figure 1).

There was an increase of VD in DCP in the operated eyes, although it was not significant; similarly, we did not find any significant correlation between vascular density of DCP and VA.

Compared to healthy fellow eyes, there was no significant difference in mean FAZ. An inverse correlation was found between FAZ area and central macular thickness (CMT) at the first and at the third month of follow up (FU) (first month: r −0.56, *p* < 0.05; third month: r −0.64, *p* < 0.05). Moreover, the FAZ area was found to be negatively correlated with foveal SCP VD and foveal DCP VD from baseline until the third month (fSCP: baseline: r −0.79, *p* < 0.05; first month: r −0.76, *p* < 0.05; third month: r −0.81, *p* < 0.05; fDCP: baseline: r −0.81, *p* < 0.05; first month: r −0.78, *p* < 0.05; third month: r −0.72, *p* < 0.05).

#### 3.2.2. “Macula-OFF” Group

The wSCP vascular density appeared to increase during follow-up, but without statistical significance. The vascular density parameters regarding the foveal and parafoveal superficial plexus and the deep capillary plexus did not have any statistically significant progression. Compared to fellow eyes, the postoperative SCP and DCP VD did not reveal any significant difference.

The FAZ area did not significantly change during follow-up. It showed a linear correlation with BCVA at the first and sixth months after surgery—the bigger the FAZ, the lower the visual acuity (*p* < 0.05). No significant difference was found in mean FAZ between healthy and affected eyes.

An inverse correlation was found between FAZ area and CMT from baseline until the third month of FU (baseline: r −0.60, *p* < 0.05; first month: r −0.56, *p* < 0.05; third month: r −0.63, *p* < 0.05). Moreover, the FAZ area was found to be negatively correlated with foveal SCP VD and foveal DCP VD at the first, third, and sixth months of FU (fSCP: first month: r −0.75, *p* < 0.05; third month: r −0.81, *p* < 0.05; sixth month: r −0.81, *p* < 0.05; fDCP: first month: r −0.84, *p* < 0.05; third month: r −0.81, *p* < 0.05; sixth month: r −0.91, *p* < 0.05).

Comparing macula-ON and macula-OFF RRD eyes, we did not find differences between the two groups in OCTA parameters.

**Figure 2:**
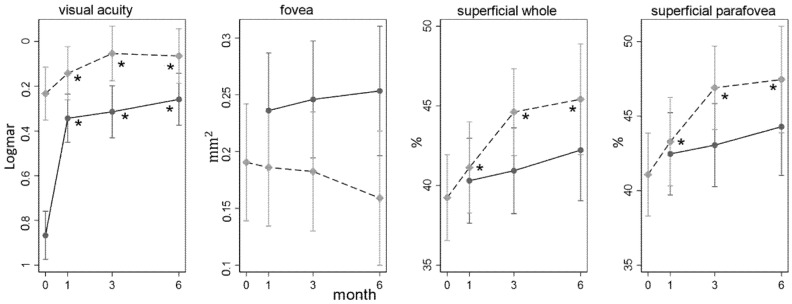
Parameter progression during follow-up. In grey, the macula-ON group; in black, the macula-OFF. Fovea: FAZ area; superficial whole: whole superficial capillary plexus density; superficial parafovea: parafoveal superficial capillary plexus density. *: a significant difference with *p* < 0.05 was found from baseline.

In Figure 2, parameter progression of significant data is shown.

#### 3.2.3. Surgical Techniques

**Figure 3:**
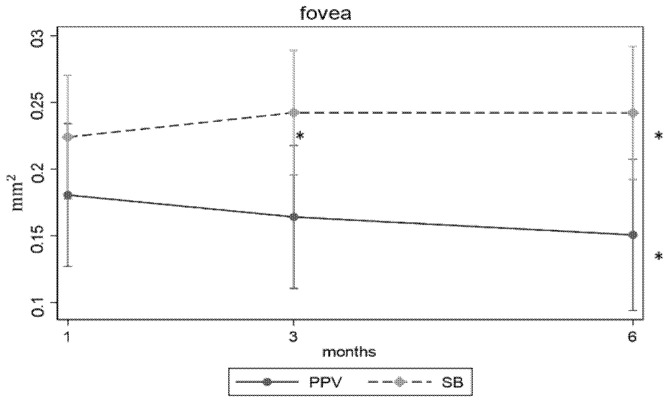
FAZ area parameter progression during follow-up with relation to surgical techniques. PPV: pars plana vitrectomy, SB: scleral buckle, fovea: FAZ area. *: a significant difference with *p* < 0.05 was found from the first month.

An analysis between PPV and SB on OCTA parameters was conducted. A reduction in the FAZ area at 6 months in PPV group was found (coefficient (coef.) −0.03, *p* < 0.05) and an augmentation in the FAZ area at 3 and 6 months in SB group was found: coef. 0.03, *p* < 0.05, and coef. 0.05, *p* < 0.05, respectively. Differences among the two groups were not significative (Figure 3).

## 4. Discussion

In this study, we evaluated the changes in the superficial and deep vascular plexi and FAZ area using OCTA, as well as their correlations with the functional outcomes in patients affected by RRD involving or not involving the macula (“macula-ON” or “macula-OFF”) operated using PPV or scleral buckle.

Over time, the retinal vasculature during RRD and after surgery was evaluated using several methods to assess the vascular alterations and to relate them to retinal function, such as BCVA. Several studies have identified the important role of the retinal perfusion in the pathophysiology of RRD and its correlation with the recovery of retinal function after surgery.

We observed that the macular involvement is the principal factor correlated with the final visual prognosis—macula-OFF patients had a lower final visual acuity than macula-ON patients. Nevertheless, we searched for other parameters using OCTA to evaluate if they were correlated with the final visual recovery or not.

In our study, macular VD in SCP gradually recovered following successful macula on RRD repair by PPV or SB (Figure 2).

Indeed, in the macula-ON group, the preoperative VD wSCP showed a significant lower value compared to the fellow eye; at 6 months, this difference disappeared. The VD wSCP had a significant linear progression over the 6 months follow-up (coef. 1.12; *p* < 0.001).

Furthermore, in the macula-ON group, we found that the patients with worse baseline visual outcome appeared to have a lower baseline VD whole and parafoveal SCP.

**Figure 4:**
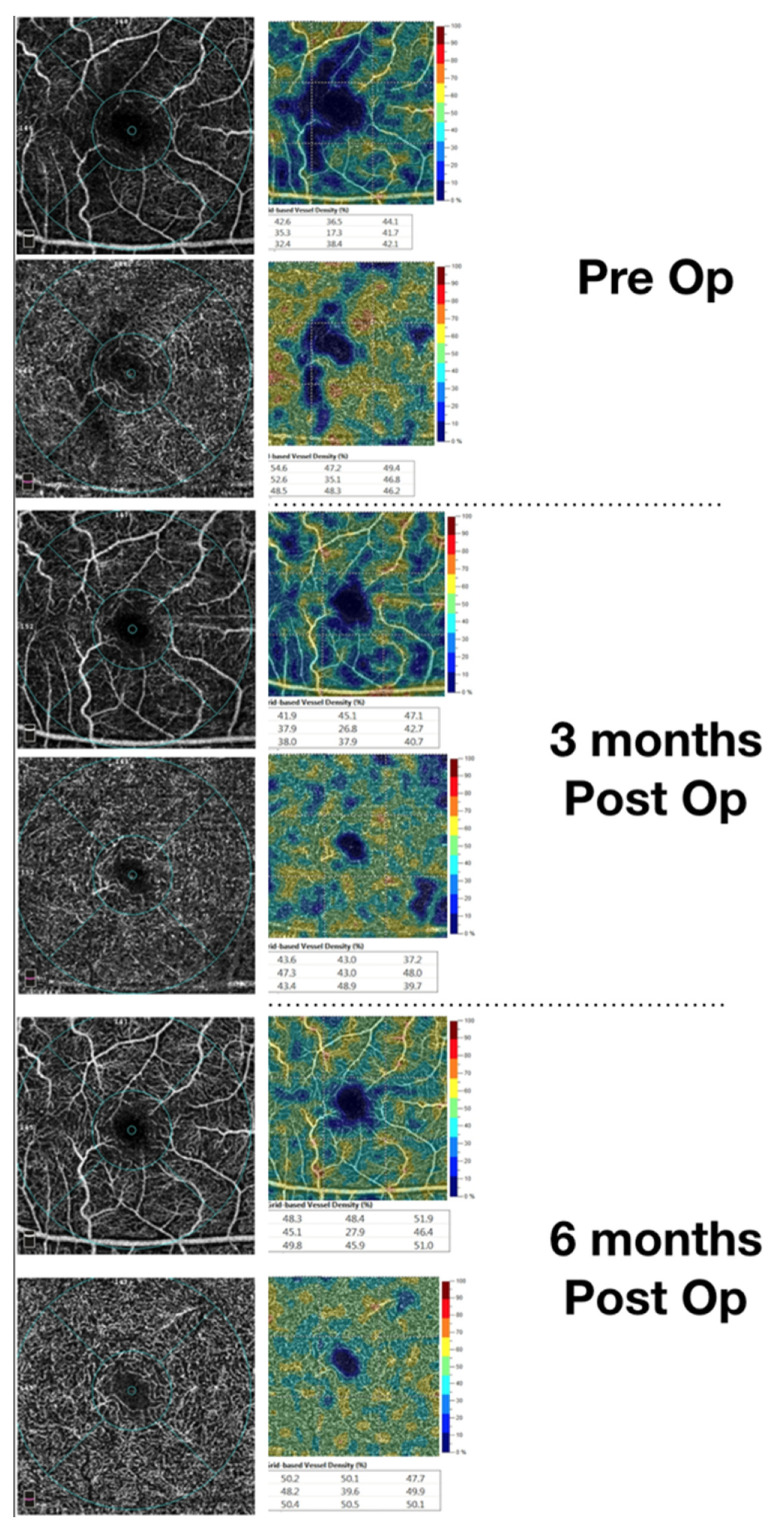
Restoration of the vascular density of the superficial vascular plexus during follow-up in a macula on patient. The upper image shows a rarefaction of the superficial vascular plexus (as shown by the vascular density (VD) map on the right). The lower image shows that a restoration can be achieved and noticed by OCTA.

Our findings confirm that even if a RRD does not detach, the macula and early reduction of the macular superficial vascular density is detectable, with a recovery at 6 months of follow-up. We could consider that the increased peripheral vascular resistances induced by the presence of RRD may cause a blood slowdown in the SCP (Figure 4).

Previous papers using fluorescein angiography reported a reduced and slow retinal blood flow in patients affected by retinal detachment due to an increase of the peripheral resistances [6]. Another report using scanning laser Doppler flowmetry demonstrated a reduced flow in retinal microcirculation in the macular area in rhegmatogenous retinal detachment (RRD) patients without macular involvement; the reduction was dependent on the extent of the RRD, and the reduction was recovered after 1 month [7]. Preoperative central retinal artery (CRA) hemodynamic parameters evaluated using a color-Doppler ultrasound device also showed worse CRA flow parameters in RRD eyes, which were influenced by preoperative RRD duration [8].

However, these studies on retinal vasculature did not assess the vascular flow in the different retinal plexuses. The advantage of using OCTA is its capacity to separately evaluate the distinct plexi that can be differently involved by the pathological processes.

Bonfiglio et al. [9], using OCTA, found a lower parafoveal deep capillary plexus density in macula-ON RRD eyes that is related postoperatively to BCVA.

We did not find significant differences in DCP VD, in contrast with recent papers—we could not exclude its absence due to the low sample of our study, even if our study design could evaluate vascular density changes that occur early after the reattachment and maybe can be transient.

We can hypothesize that in a recent onset disease such as retinal detachment, and even more so in RRD without macular involvement, that the SCP is the most affected plexus because it may be the first vascular layer involved by a rapid increase in vascular resistances induced by RRD. The SCP could develop a stronger and faster contraction in comparison with DCP thanks to its greater density in arterioles and in smooth muscle. The DCP, even if more vulnerable to hypoxic damage, may be later involved. Meanwhile, our knowledge in DCP modifications is mostly based on chronic pathologies such as diabetic retinopathy, where the supposed pathogenic factors act in a slower manner than in RRD. Moreover, in our study, we assessed the vascular density in recent onset RRD that had undergone surgical intervention in a brief time, and we performed the examination in an early phase after surgery. We could hypothesize that these inclusion criteria may partially explain our OCTA findings related to different behavior of retinal capillary plexi.

Other important factors involved in VD reduction could be the endothelin-1 release and the role of the Müller cells in the pathological process. Roldan-Pallares et al. [10] found significant levels of the endothelin-1, a vasoconstrictive peptide, in subretinal fluid (SRF), probably also synthetized by endothelial and glial cells; its action causes a vasoconstriction on retinal microvasculature and a consequent reduction in blood flow [11]. Endotelin-1 has a role also on retinal Müller cells [12], favorizing their activation in detached and nondetached retinal regions [13]. Müller cells activation consists of gliosis, cellular hypertrophy, and subretinal fibrosis [14], and could affect retinal vasculature in the absence of macrostructural changes [15].

Additionally, a recent study [16] found that in cases of hypoxia, aberrant oxygen metabolism could rise the metabolic demands from the retinal vasculature, instead of choroidal oxygen extraction. Thus, in the case of RRD, hypoxia occurs in the detached retina and oxygen extraction is taken from retinal vasculature; in parallel, endotelin-1 also provides vasoconstriction in the attached retina, explaining the reduction in VD found in OCTA investigation. This aberrant mechanism could explain a certain grade of retinal and macular ischemia, even reversible; in fact, our study and literature [9,17,18] showed an increase in VD over time. Moreover, macular involvement during RRD could affect visual acuity and a lower VD could explain a reduction in BCVA, as we found. However, these mechanisms are already uncertain and proper further studies should be performed.

In the macula-OFF group, the wSCP vascular density appeared to increase during follow-up, but without statistical significance. The vascular density parameters regarding the foveal and parafoveal superficial plexus and the deep capillary plexus did not have any statistically significant progression. Compared to fellow eyes, the postoperative SCP and DCP VD did not reveal any significant difference.

We have to take into account the fact that in the macula-OFF group we did not find any significant differences in SCP and DCP vascular density. Possible reasons for the discrepancies between our results and other studies may be the differences in terms of baseline characteristics of study subjects (including eyes treated with vitrectomy or scleral bucking, and different inclusion and exclusion criteria); different OCTA parameters, including VD from different regions of interest (i.e., whole image, foveal, parafoveal, perifoveal); number of patients included; and follow-up period.

**Figure 5:**
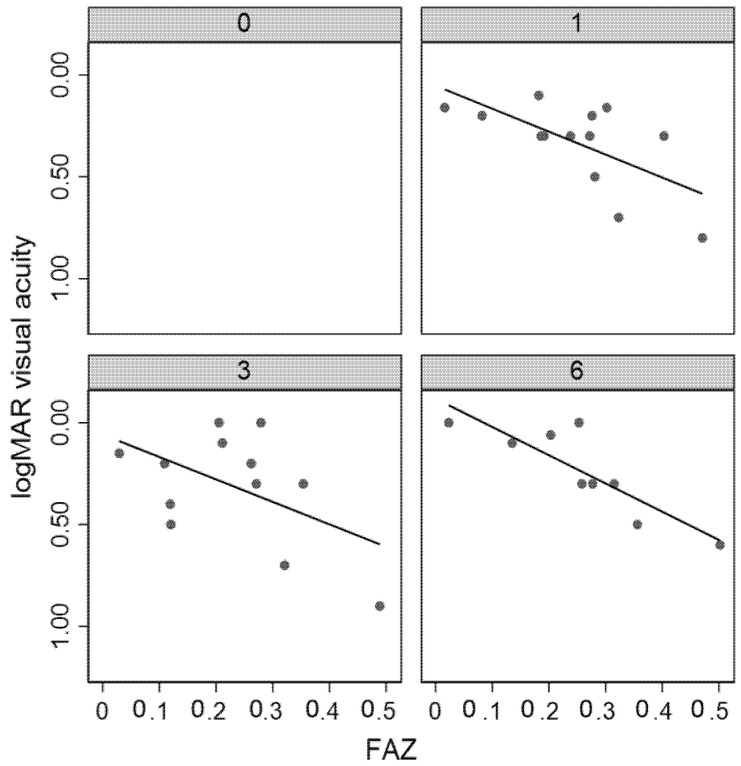
Foveal avascular zone (FAZ) correlation to visual acuity during follow-up.

Regarding the FAZ changes in the macula-OFF group (Figure 2), we could not find any significant progression in its change during follow-up nor any difference among the affected and the fellow FAZ. On the other hand, in agreement with literature, we noticed that after retinal reattachment, FAZ was inversely correlated to the BCVA at months 1 and 6—the larger the FAZ, the worse the visual acuity (Figure 5).

OCTA can measure the size of the FAZ dimension reproducibly to generate quantitative vascular information [19]. The FAZ is the central region in the retina that is entirely devoid of vessels, including capillaries. This region has the highest cone density, and it is an area of high metabolic activity. In healthy eyes, the FAZ size varies but does not seem to influence vision [20]. However, FAZ size and VA may be correlated with eyes with retinal disease. A group of authors [21], using scanner laser ophthalmoscopy, and another group [22], using fluorescein angiography, showed that the FAZ area was larger in the affected eyes than in the control eyes in central retinal vein occlusion (CRVO) and branch retinal vein occlusion (BRVO), respectively. Woo and colleagues [18] support the hypothesis of a similar mechanism in retinal vein occlusion (RVO) and in RRD, both characterized by diffuse vascular occlusion and tissue hypoxia, and have supposed that, even in case of intact IS/OS junction, macular ischemia could negatively affect the VA. A larger FAZ could be a representation of this mechanism.

FAZ area in some cases of this group appeared to be very small—no ERMs were found; we hypothesize that a small FAZ could be the result of a macular displacement after retinal reattachment. In addition, internal limiting membrane (ILM) peeling was performed during PPV, adding a potential causal factor of FAZ reduction in this group [23]. Further studies are needed to confirm this hypothesis.

A reduction in the FAZ area at 6 months in the PPV group was found; moreover, an augmentation in the FAZ area at 3 and 6 months in the SB group was found (Figure 3).

Our hypothesis to explain these findings are as follows: in SB patients, the FAZ enlargement can be attributed to the macula off group. A FAZ enlargement was found in these patients that was probably produced by the ischemia caused by the detachment, as it happens in patients affected by diabetes and retinal vein occlusion. On the other hand, patients who underwent PPV seem to follow an inverse trend with a gradual reduction of the FAZ. This can be explained by the fact that all the patients enrolled in the PPV group underwent an ILM peeling. Some papers [24,25] suggest that a lack of FAZ area increase after surgery can be caused by a collateral effect of the ILM peeling. One explanation is that the ILM may have some intrinsic forces stretching the retina centrifugally, and its peeling may remove such forces, leading to a centripetal movement. The second hypothesis is that the structural changes to the Muller cells, caused by damage during ILM removal, may influence the inner retinal movement. These cells also act as a scaffold that stretches the macula outwards; the removal of their footplates, which are anchored to the ILM, stops this action, and the macula may move inwards.

Recently, the choriocapillary layer (CCP) was investigated in patients undergoing surgery for RRD repair [26,27]. In macula-OFF patients with an outer retinal defect, CCP vascular density was significantly correlated to VA, hypothesizing that choroidal vascularization can affect the IS/OS segment trophism. Further studies are needed to investigate if a possible correlation between CCP vessel density and FAZ area is present in macula-OFF RRD.

Our study has several limitations. First, the sample size was relatively small. Second, we did not evaluate the height and volume of the macular detachment, which might have affected the postoperative macular VD results. Third, the follow-up period was relatively short.

Operative time and retinal detachment extension can influence plexi recovery; however, we did not consider these parameters in our study. This will certainly be a point to investigate in a larger sample of patients.

A further prospective randomized study with a larger number of patients and a longer observation period is warranted to confirm these results.

In conclusion, OCTA is a technique that can assess, in a rapid and noninvasive way, the macular microvasculature in patients affected by RRD. In macula-ON patients, using OCTA, macular SCP VD was revealed to be reduced in comparison with the fellow eye, and it recovered over time. The hypothesis of a remitting hypoxic mechanism may be suggested; nevertheless, further studies should be warranted to confirm this hypothesis.

In macula-OFF patients, a larger FAZ area was found to be related to a worse VA, is the case in diabetes and in RVO.

The influence of different surgical techniques on macular microvasculature requires further studies in order to assess its impact on retinal microvasculature.

## Figures and Tables

**Table 1:** Baseline demographics and ocular characteristics.

	Macula-ON	Macula-OFF
Numerosity	15 (4 F, 11 M)	18 (9 F, 9 M)
Age	59.25 ± 13.38 (min 27, max 77 yo)	61.11 ± 10.04 (min 47, max 79 yo)
Surgical technique	7 SB, 8 PPV	8 SB, 10 PPV

Abbreviations: “F” female, “M” male, “yo” years old, “SB” scleral buckle, “PPV” pars plana vitrectomy.

**Table 2:** Optical coherence tomography angiography and clinical parameters of the macula-ON group.

Macula-ON	wSCP (%)	fSCP (%)	pfSCP (%)	wDCP (%)	fDCP (%)	pfDCP (%)	FAZ Area (mm^2^)	BCVA (LogMar)	CMT (μm)
Baseline	39.24 ± 6.64	19.54 ± 7.10	41.08 ± 6.97	48.40 ± 4.09	36.58 ± 5.75	50.77 ± 4.42	0.24 ± 0.18	0.23 ± 0.26	289.58 ± 62.95
1st month	41.01 ± 5.26 *	22.38 ± 6.92	43.20 ± 5.83 *	48.70 ± 4.40	37.43 ± 5.76	50.73 ± 4.58	0.19 ± 0.07	0.14 ± 0.13 *	298.13 ± 39.82
3rd month	44.61 ± 4.40 *	23.01 ± 5.25	46.9 ± 3.84 *	49.39 ± 4.92	38.11 ± 5.04	51.12 ± 5.01	0.18 ± 0.09	0.05 ± 0.09 *	309.53 ± 55.16
6th month	44.87 ± 5.12 *	23.27 ± 5.9	46.82 ± 5.90 *	50.83 ± 4.15	37.10 ± 5.11	52.43 ± 5.44	0.18 ± 0.09	0.06 ± 0.11 *	313.18 ± 57.15
Fellow eyes	44.54 ± 4.30 *	20.23 ± 7.47	47.10 ± 4.72	49.25 ± 3.88	36.64 ± 7.62	50.70 ± 3.71	0.22 ± 0.09	0.11 ± 0.13 *	272.47 ± 32.65

wSCP: whole superficial capillary plexus; fSCP: foveal superficial capillary plexus; pfSCP: para-foveal superficial capillary plexus; wDCP: whole deep capillary plexus; fDCP: foveal deep capillary plexus; pfDCP: para-foveal deep capillary plexus; FAZ: foveal avascular zone; BCVA: best corrected visual acuity; CMT: central macular thickness. *: a significant difference with *p* < 0.05 was found from baseline.

**Table 3:** Optical coherence tomography angiography and clinical parameters of the macula-OFF group.

Macula-OFF	wSCP (%)	fSCP (%)	pfSCP (%)	wDCP (%)	fDCP (%)	pfDCP (%)	FAZ Area (mm^2^)	BCVA (LogMar)	CMT (μm)
Baseline	n/a	n/a	n/a	n/a	n/a	n/a	n/a	0.87 ± 0.34	n/a
1st month	39.98 ± 6.17	22.34 ± 9.79	42.20 ± 6.12	49.62 ± 4.63	36.15 ± 8.16	51.82 ± 6.83	0.25 ± 0.12	0.34 ± 0.25 *	275.23 ± 62.28
3rd month	40.38 ± 5.33	22.03 ± 8.9	42.58 ± 5.68	47.97 ± 4.26	35.15 ± 8.19	50.31 ± 4.36	0.24 ± 0.12	0.33 ± 0.31 *	262.06 ± 54.53
6th month	42.15 ± 5.71	23.25 ± 11.74	44.13 ± 5.21	49.69 ± 6.21	35.68 ± 8.89	52.17 ± 6.72	0.26 ± 0.14	0.26 ± 0.27 *	270.56 ± 60.35
Fellow eyes	37.16 ± 15.75	17.77 ± 9.82	38.83 ± 17.81	42.32 ± 18.70	35.23 ± 9.55	51.20 ± 6.54	0.27 ± 0.16	0.09 ± 0.17 *	279.52 ± 35.38

wSCP: whole superficial capillary plexus; fSCP: foveal superficial capillary plexus; pfSCP: para-foveal superficial capillary plexus; wDCP: whole deep capillary plexus; fDCP: foveal deep capillary plexus; pfDCP: para-foveal deep capillary plexus; FAZ: foveal avascular zone; BCVA: best corrected visual acuity; CMT: central macular thickness; n/a: not applicable. *: a significant difference with *p* < 0.05 was found from baseline.
